# Resistance training and caloric restriction prevent systolic blood pressure rise by improving the nitric oxide effect on smooth muscle and morphological changes in the aorta of ovariectomized rats

**DOI:** 10.1371/journal.pone.0201843

**Published:** 2018-08-22

**Authors:** Anderson Diogo de Souza Lino, Daiana Vianna, Jorge Camargo Oishi, Markus Vinicius Campos Souza, Leandro Dias Ruffoni, Cecília Tardivo Marin, Lucimar Retto da Silva de Avó, Sérgio Eduardo de Andrade Perez, Gerson Jhonatan Rodrigues, Júlio Tirapegui, Gilberto Eiji Shiguemoto

**Affiliations:** 1 Laboratory of Exercise Physiology, Department of Physiological Sciences, Center of Biological and Health Sciences, Interinstitutional Post-Graduate Program in Physiological Sciences, Federal University of São Carlos – UFSCar, São Carlos, Brazil; 2 Laboratory of Nutritional Biochemistry, Department of Food and Experimental Nutrition, University of São Paulo - USP, São Paulo, Brazil; 3 Laboratory of Pharmacology, Department of Physiological Sciences, Center of Biological and Health Sciences, Interinstitutional Post-Graduate Program of Physiological Sciences, Federal University of São Carlos – UFSCar, São Carlos, Brazil; 4 Physical Education Course, Department of Sports Science, Post-Graduate Program in Physical Education, Federal University of Triângulo Mineiro, Uberaba, Brazil; 5 Laboratory of Neuroendocrinology, Department of Physiological Sciences, Center of Biological and Health Sciences, Interinstitutional Post-Graduate Program of Physiological Sciences, Federal University of São Carlos – UFSCar, São Carlos, Brazil; 6 Medical Department, Center of Biological and Health Sciences, Federal University of São Carlos – UFSCar, São Carlos, Brazil; Max Delbruck Centrum fur Molekulare Medizin Berlin Buch, GERMANY

## Abstract

In this study, we investigated the effects of resistance training (RT), caloric restriction (CR), and the association of both interventions in aortic vascular reactivity and morphological alterations, matrix metalloproteinase-2 (MMP-2) activity, insulin resistance and systolic blood pressure (SBP) in ovariectomized rats. Fifty female Holtzman rats were subjected to ovariectomy and Sham surgery and distributed into the following groups: Sham-sedentary, ovariectomized-sedentary, ovariectomized-resistance training, ovariectomized-caloric restriction, and ovariectomized-resistance training and caloric restriction groups. RT and 30% CR protocols were performed for 13 weeks. Analyses were conducted to evaluate the following: acetylcholine and sodium nitroprusside-induced relaxation of aortic rings, MMP-2 activity, insulin tolerance test, highlighting of the aorta wall cross-sectional area by hematoxylin-eosin stain, aorta vessel remodeling and SBP. We observed that ovariectomy decreased the potency of dependent and independent endothelium relaxation and MMP-2 activity, prevented insulin resistance, promoted aorta vessel remodeling in the cross-sectional area, and promoted the media-to-lumen ratio, the collagen content, and the alteration of the structure and elastic fibers of the vessel. The effects of the ovariectomy could contribute to SBP increases. However, the association of exercise and diet improved the relaxation potency in dependent and independent endothelium relaxation, elevated MMP-2 activity, ameliorate insulin sensitivity, increased the aorta cross-sectional area and media-to-lumen ratio, decreased collagen content and promoted histological parameters of the aorta vessel wall, preventing the increase of SBP. Conclusion: Our study revealed that the RT and CR separately, and even associatively, improved vascular function, activated MMP-2, and produced a beneficial hypertrophic remodeling, preventing the elevation of SBP in ovariectomized rats.

## Introduction

Estrogen hormones play important role in maintaining aortic wall relaxation properties and function, in addition to controlling blood pressure. In estrogen deprivation, as in the condition of menopause, the properties of the aortic wall are impaired, which could result in systolic blood pressure (SBP) elevation [1, 2]. In addition, in this condition, insulin resistance is a pathogenic factor which may be related in modulating vascular tonus, contribute to decreasing the relaxation of the vessel [3], and play a role in the emergence of hypertension. In hypertension, arteries lose elasticity and accumulate connective tissue, such as collagen [1, 2]. These factors can alter the mechanical properties of the aortic wall and its capacity to distend [1], promoting SBP elevation and leading to cardiovascular disease [4].

One proteolytic enzyme that is involved in the modification of aortic structures is the matrix metalloproteinase-2 (MMP-2) [1]. This enzyme has a relevant effect on endothelium and smooth muscle, which may be important in the early stages of vascular remodeling in order to maintain blood flow to various organs [5]. MMP-2 also exerts an effect on collagen and on the elastic fibers, which are the structural components of the aorta [2]. Ovariectomy (OVX) is able to reduce MMP-2 activity in the thoracic aorta with a significant increase in collagen accumulation. This effect has been reverted by estrogen replacement, showing an estrogen role in vascular collagen accumulation by modulating MMP-2 activity [6]. In addition, with aging, the activity of MMP-2 increases with the decrease in the quantity and functionality of elastic fibers (elastin) and the increase in the collagen content in the aortic artery, which is another factor that can lead to hypertension in the absence of estrogen hormones [2, 7]. The elastic fiber can be degraded by MMP-2, resulting in a change in ECM homeostasis modification, collagen deposition and arterial stiffness [2]. Clinical reports indicated a decreased MMP-2 protein expression in plasma from patients with hypertension [8]. The decreased MMP-2 activity in the hypertensive patients is involved in the vascular remodeling in the hypertension condition, showing a potential link relating to vascular compliance and hypertension in menopause by the increased of collagen content as well as decreased MMP activity. Thus, the artery remodeling may be the major causes of the development of ECM accumulation and hypertension [9].

A method to study the effects of estrogen deprivation is the OVX, a good model for mimicking human ovarian hormone loss, condition that observed in human menopause. This model allows the development of new types of treatments and predicts the results of therapeutic interventions in humans [10]. In OVX rats, exercise training [11, 12] and CR [13] could positively modulate aortic wall remodeling and vascular resistance, which together could prevent the rise in SBP. However, most of the molecular and morphological structure and function alterations involved in such beneficial effects of RT and CR in the OVX rats are still unknown.

In addition, the study of possible morphofunctional alterations in aorta arteries is very important because this artery is responsible for storing half of the left ventricular ejection volume in the systolic phase. In the diastole, the elastic forces of the aortic wall propel this volume to the peripheral circulation. This results in normal continuous peripheral blood flow [1]. This aortic function provides a reduction of left ventricular afterload and improvement of coronary blood flow and left ventricular relaxation. Impaired aortic function may lead to increased elastic resistance of the vessel resulting in increased blood pressure in postmenopausal women, that can leading to hypertension [1]. The impairment of aorta function, as in aortic stiffness and endothelial dysfunction [14], is associated with OVX rats [15].

However, in OVX rats, the influence of RT, CR, and their association in aorta morphological structure and function, MMP-2 activity and SBP is not yet clear. Our hypotheses are that the RT, CR and the association of RT and CR can mitigate or even prevent the deleterious effects caused by the absence of ovarian hormones promoted by OVX in the aorta morphological structure and function, which could prevent the SBP elevation in OVX rats. Thus, the aim of the present study is to investigate the effects of RT, CR, and the association of RT and CR on aortic vascular reactivity, morphological alterations, and MMP-2 activity, preventing the elevation of SBP in OVX rats.

## Materials and methods

### Animals

Animal protocols were approved by the Committee of Experimental Animals from the Federal University of São Carlos/UFSCar (#004/2013) and were conducted in accordance with the principles outlined by the Brazilian College of Animal Experimentation (COBEA) and the Guide for the Care and Use of Laboratory Animals, published by the US National Institute of Health (NIH, 8^th^ Edition, 2011).

Female offspring of Holtzman albino rats (*Rattus novergicus var*. *albinus*, *Rodentia*, *Mammalia*, from the breeding colony of the State University of São Paulo/UNESP, Araraquara, Brazil) were housed at a controlled temperature (22±2°C), relative humidity of 55 ± 10%, with 15 to 20 air changes per hour, and a 12 h light/dark cycle, with light from 07:00 AM to 07:00 PM. When the animals reached the weight of 250 g, for adaptation before OVX, all of them were submitted to a reversed light cycle of 12 h light/dark, with light from 07:00 PM to 07:00 AM.

### Experimental groups

Animals (*n* = 50) were randomly distributed into two principal groups: A) Sham and B) ovariectomized (OVX). These two principal groups were further subdivided into five groups (n = 10): 1) Sham-sedentary (Sham-SED); 2) ovariectomized-sedentary (OVX-SED); 3) ovariectomized-resistance training (OVX-RT); 4) ovariectomized-caloric restriction (OVX-CR); and 5) ovariectomized-resistance training associated with caloric restriction (OVX-RT+CR).

### Surgical procedures

Animals were exposed to the surgical procedures described above, and all that underwent surgery had 10 days of recovery until the onset of the RT and CR protocols.

#### Ovariectomy (OVX) and Sham surgery

Rats were submitted to OVX procedures under an anesthetic condition (ketamine—xylazine, 61.5–7.6 mg/100 g) when they reached 275 g. A small incision was performed bilaterally (1.0–1.5 cm) through the skin and muscle layer between the last rib and the thigh parallel to the line of the animal body. The peritoneal cavity was opened, and a bandage was held below the fimbria. The ovaries were removed, and the incisions in the skin and muscles were sutured. Animals were medicated for 3 days with antibiotics (penicillin-streptomycin, 5 mg/kg). Sham surgery was performed using the same procedures of OVX, except for the ligation below the fimbria and the removal of ovaries, which were only exposed and returned to the original location [10, 16]

### Resistance training protocol (RT)

We used the RT protocol from Hornberger and Farrar [17], adapted by previous work [18]. The RT protocol was performed on a vertical ladder (1.1 m x 0.18 m, 2 cm grid, 80° incline) and a housing chamber (20 cm^3^), where the rats were allowed to rest. The apparatus consisted of a falcon tube with a fishing sinker tied to the proximal portion of the rat’s tail by a self-adhesive foam strip, as workload. Familiarization of rats with the protocol was performed in three sessions. The rats were considered adapted to the protocol by climbing the vertical ladder three consecutive times, from the bottom to the housing chamber, with two min of rest in the house chamber, without stimulus. The load apparatus was attached to their tails with no weight.

#### Maximal workload determination and RT session

The initial climb consisted of a carrying load that was 75% of the animal’s body mass, and additional 30 g weights were added until the load did not allow the animals to climb the entire length of the ladder. The highest load successfully carried over the whole length of the ladder was considered as the rat’s maximal carrying capacity for that training session.

RT sessions were performed with 72 h of intervals; with four climbs with 65, 85, 95, and 100% of the rat’s previous maximum workload. During the subsequent ladder climbs, an additional 30 g load was added until a new maximal carrying capacity was determined. The animal was allowed at most five extra climbs (if the rat reached the last climb) before the 100% workload climb.

### Caloric restriction intervention (CR)

The amount of food provided to each rat within the CR groups was calculated before starting the experimental protocol when animals had free access to food. The average daily consumption of the rats was observed for 30 days before starting the experiment, and 30% of each rat’s consumption was restricted. OVX rats with CR (OVX-CR; OVX-RT+CR) received 70% of their food consumption of the previous experimental protocol. Diets were fed twice a day, in the morning (10:00 AM) and after training (02:00 PM).

The CR diet was adjusted to avoid nutritional deficiencies, increasing the nutrients in proportion to food restriction [19, 20]. The diet composition used in the experiment was in accordance with the AIN-93M [21].

### Measurement of systolic blood pressure (SBP) and heart rate (HR) by plethysmography

**Fig 1:**
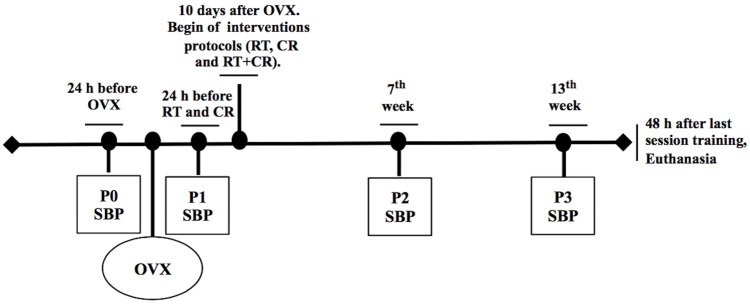
Scheme of the experimental period. OVX = Ovariectomy procedures; SBP = Systolic blood pressure measurements; P0 = 24 h before OVX; P1 = 10 days after OVX and 24 h before RT and CR starting protocols; P2 = seventh week after RT and CR protocol starting and P3 = 24 h before euthanasia.

SBP was measured four times during the experimental period (Fig 1): 24 h before OVX (P0), ten days after OVX and 24 h before starting RT and CR protocols (P1), seven weeks after starting RT and CR protocols (P2), and 24 h before euthanasia (P3). The SBP was performed with animals that were awake by tail plethysmography, as previously described [22].

The HR was analyzed on a beat-to-beat recording of each animal at the same time of SBP, in the initial (after OVX) and in the final experimental period.

### Insulin tolerance test (ITT) and plasma glucose decay rate constant (kITT) analyses

Animals were submitted to ITT after a 12 h fast. Blood glucose was determined by the glucometer, Accu-Chek(^®^) Performa (Roche Diagnostics) in blood samples collected from the caudal vein at times 0, 5, 10, 15, 20, 25, and 30 min after insulin administration (0.75 U/kg body mass; Humulin R, Eli Lilly and Company, Indianapolis, Indiana, USA) [23]. Values obtained between 5 and 30 min were used to calculate the plasma glucose decay rate constant (kITT), by means of the decay curve analysis (by the GraphPad Prism version 4.0 for Windows program). This test was performed at the end of the experimental protocol.

### Tissue dissection

The thoracic aorta was excised after thoracotomy and was isolated and cleaned from the adipose and connective tissue. The aorta rings (5 mm long) were immediately separated into three portions to describe the analysis in the sequence. The uterus was dissected, weighted and normalized by tibial length (TL); uterus mass /tibia length ratio (U/TL ratio).

### Vascular reactivity in aorta

To perform the vascular reactivity experiment, an isometric Resistance myograph system [24] (model 610 DMT-USA, Marietta, GA) and a PowerLab8/SP data acquisition system (AD Instruments Pty Ltd., Colorado Springs, CO) to record data were used.

Thoracic aorta rings were isolated and placed in a Krebs solution. The aortas were carefully dissected and mounted (with intact endothelium and denuded endothelium) as ring preparations (≅5 mm in length) and placed in bath chambers (5 mL) containing a pH 7.4 Krebs solution at 37°C, which was continuously bubbled with 95% O_2_ and 5% CO_2_. The aortic rings were submitted to a tension of 1.5 g, which was readjusted after every 15 min during a 60 min equilibration period, before addition of the given drug. An optimal basal tension of 1.5 g in the aortic rings from both Sham and OVX groups was previously standardized by exposing the aorta rings to 90 mM KCl under various resting tensions (0.25–2.5 g), as previously described [22].

#### Protocol

The endothelial integrity of the aorta rings was assessed by the degree of relaxation induced by 1 μM/L acetylcholine (ACh) in the presence of contractile tone induced by phenylephrine (0.1 μM/L). The ring was discarded if relaxation with acetylcholine was lower than 80% in the rat aorta. After the endothelial integrity test, aortic rings were precontracted with phenylephrine (0.1 μM). When the plateau was reached, concentration—effect curves to acetylcholine (0.1 nM to 0.1 mM) were constructed. The potency (pD2) and the maximum relaxant effect were evaluated. The preparation of the ring without endothelium was performed by gently rubbing the intimal surface of the ring with a metal rod to remove it. The endothelium removal procedure was verified by the inability of acetylcholine (10–6 M) to relax arteries precontracted with phenylephrine (0.1 μM/L) [22].

### Determination activity of MMP-2 in aorta by gelatin zymography

Frozen aliquot aorta was macerated in liquid nitrogen and incubated in (10 μL/mg of tissue) extraction buffer (10 mM cacodylic acid, pH 5.0; 0.15 M NaCl; 1 μM ZnCl_2_; 20 mM CaCl_2_; 1.5 mM NaN_3_; 0.01% Triton X-100 [v/v]), at 4°C overnight for 22 h and centrifuged for 20 min (13,000xg at 4°C). Thirty micrograms of total protein measured with the BCA Protein Assay Kit (Pierce, Rockford, IL, USA) were loaded and applied in each lane of sodium dodecyl sulfate (SDS)–10% polyacrylamide gels prepared with 1 mg/mL gelatin. After electrophoresis, the gels were washed twice for 20 min in a 2.5% Triton X-100 to remove the SDS. The gels were rinsed and incubated in a buffer substrate (50 mM Tris-HCl, pH 8.0; 5 mM CaCl_2_; 0.02% NaN_3_) at 37°C for 20 h. The gels were stained with Brilliant blue for 60 min and destained with an acetic acid:methanol:water (1:4:5) mixture for visualization of the activity bands. The gels were photographed in a Molecular Imager^®^ Gel Doc^™^ XR+ System. Densitometry quantitative analyses were performed using the Gene Tools version 3.06 software (Syngene, Cambridge, UK) [16].

### Morphometric collagen content analysis of the aorta wall

The 5 mm thoracic aortic rings were imbibed in fixing solution of 10% formaldehyde for 24 h at room temperature (±25°C). Sections of 5 μm thickness were cut in a microtome (Microm HM 340e, Walldorf, Germany) with low-profile razors (Leica) and collected on glass slides (Knittel^®^, Germany). Samples were stained with hematoxylin and eosin (H&E), Trichrome-Masson and Picrosirius Red. Semiserial cuts with 8 sections per rat and 8 rats per group were performed. Images of the aorta rings’ sections for H&E and Trichrome-Masson were obtained by a Pannoramic Digital Slide Scanners system to capture the entire slide digitally, in high-resolution images with a 40× objective. Images were measured by the Pannoramic Viewer image analysis software (3DHISTECH, Ltd.). External (Ae) and internal (Ai) areas were determined by the calculation of the internal and external elastic membrane. To calculate external (ED) and internal (ID) diameter, the equations used were the square root of 4Ae/π and 4Ai/π, respectively. Media thickness (M) was calculated as the difference between ED and ID divided by 2 (M = ED—ID/2). The media-to-lumen ratio (M/L) was also calculated [25]. The media cross-sectional area (CSA) was calculated by subtracting the lumen internal area (Ai) from the external area (Ae) (CSA = Ae—Ai) [26, 27].

The number of lamellae was counted in four fields, located at 0°, 90°, 180°, and 270°, and stained with Trichrome-Masson. The arithmetic mean of the number of lamellae observed in each field was calculated. To identify the degree of histological change of the aortic wall, the histological samples were fit into three different categories: category 1 –well-defined endothelial cells, smooth muscle cell without visible changes, linear and parallel elastic fibers, and interspersed with smooth muscle; category 2—fewer endothelial cells, fewer smooth muscle cells, and elastic fibers slightly undulated with minimal space and fewer smooth muscle cells between them; and category 3—few endothelial cells, misshapen structures, few smooth muscles, altered, wrinkled, and thick elastic fibers with minimal space and less smooth muscle between them. A subjective analysis of endothelial cells and the smooth muscle quality and quantitates was performed, and an experienced pathologist performed the elastic fiber morphology.

Collagen (I and III) content was determined as a percentage (%) of the total area in four fields as cited above, and the samples were stained with the Picrosirius Red technique and examined under polarized light. The images from aorta sections were captured and analyzed by a system composed of a video camera coupled to an Olympus model BX51 microscope, which was linked to a microcomputer with an image digitalizing plate. The program ImageJ (National Institutes of Health) was used to perform these structural analyses.

### Statistical analysis

The data were presented as the mean ± standard error of the mean (SEM). The Kolmogorov—Smirnov test was used to analyze the normality of the data. A one-way ANOVA, followed by the post hoc Tukey test (p<0.05), was used to test the significant difference among all groups. The software packages, IBM SPSS^®^ Statistics, version 20 (statistical analysis) and GraphPrism 5 (graphics) for Macintosh, were used for analyses.

## Results

### Body mass, U/TL ratio, kITT and maximal work load

In the initial procedures, the body mass (BM) of all groups did not differ. In the final experimental period, OVX promoted a significant increase in BM of sedentary animals (OVX-SED) (p<0.0001). The BM of OVX-RT (p = 0.04), OVX-CR (p = 0.007), and OVX-RT+CR (p = 0.001) showed lower values compared to that of the OVX-SED group. RT protocol reversed the gain mass induced by OVX (OVX-SED *vs* OVX-RT, p = 0.04; OVX-SED *vs* OVX-RT+CR, p = 0.001). Comparing both trained groups, we observed that the OVX-RT+CR showed lower BM values compared to that of the OVX-RT (p = 0.017). No difference was observed between OVX-CR *vs* OVX-RT+CR. In addition, the interventions were able to maintain BM values equal to those of the Sham-SED group (Table 1).

**Table 1:** Values of body mass, U/TL ratio, kITT test and maximal workload.

	Experimental Groups				
	Sham	Ovariectomized (OVX)				
	SED	SED	RT	CR	RT+CR
**BM Initial (g)**	276 ± 05	274 ± 05	292 ± 04	293 ± 06	283 ± 06
**BM Final (g)**	372 ± 14 ^b^	468 ± 13 ^a^	416 ± 16 ^b, c^	404 ± 10 ^b^	358 ± 09 ^b, d^
**U/TL (mg/cm)**	0.18 ± 0.023 ^a^	0.03 ± 0.003 ^b^	0.05 ± 0.008 ^b^	0.03 ± 0.003 ^b^	0.03 ± 0.002 ^b^
**kITT (%min**^**-1**^**)**	1.93 ± 0.35 ^b^	3.03 ± 0.16 ^a^	2.08 ± 0.39 ^b^	2.92 ± 0.48 ^a, c^	2.21 ± 0.32 ^b, c^
**Initial MW (g)**	_	_	491 ± 24	_	527 ± 19
**Week 7 MW (g)**	_	_	1,012 ± 38 *	_	1,029 ± 31 *
**Final MW (g)**	_	_	1,065 ± 40 *	_	1,095 ± 28 *

Values are presented as the mean ± standard error of mean (SEM). BM, body mass (g); MW, maximal workload; kITT, plasma glucose decay rate constant. U/TL, uterus/tibial length ratio.

Superscript letters represent significant differences between groups by a one-way ANOVA followed by post hoc Tukey test (p<0.05), a ≠ b, b ≠ c, c ≠ d.

* represents significant difference from Initial MW *vs* Week 07 MW and Final MW; and Week 07 MW *vs* Final MW.

To control surgery efficacies, the uterus mass / tibia length ratio (U/TL ratio) was measured, and as expected, it was found to be lower in all OVX groups, indicating a significant negative change in ovarian hormone concentration (Sham-SED *vs* OVX-SED, p = 0.0001). On the other hand, compared to Sham-SED, the RT and CR protocols, even RT+CR, did not avoid this effect (p = 0.0001). Between OVX groups, the U/TL ratio did not change.

Insulin resistance promoted by OVX in sedentary animals was observed when compared to that of Sham-SED (p = 0.002). RT improved insulin sensitivity in OVX-RT compared to the OVX-SED (p = 0.006) and the OVX-CR (p = 0.02) groups, with no difference compared to Sham-SED. CR was not able to improve insulin sensitivity compared to that of Sham-SED. Insulin sensitivity was improved by RT+CR in OVX animals compared to that of the OVX-SED (p = 0.01) and the OVX-CR (p = 0.04) (Table 1).

The maximal workload (MW) in all trained groups differed from week 01 (RT-protocol-beginning) to week 07 and week 13 (final RT protocol) (p = 0.001, respectively). From week 07 to week 13, we observed maximal workload stagnation in both trained groups (p>0.141). We did not observe a significant difference (p>0.964, respectively) between RT and RT+CR in OVX animals in the initial MW *vs* week 07 MW and final MW or in the week 07 MW *vs* final MW (Table 1).

### Vascular reactivity

**Fig 2:**
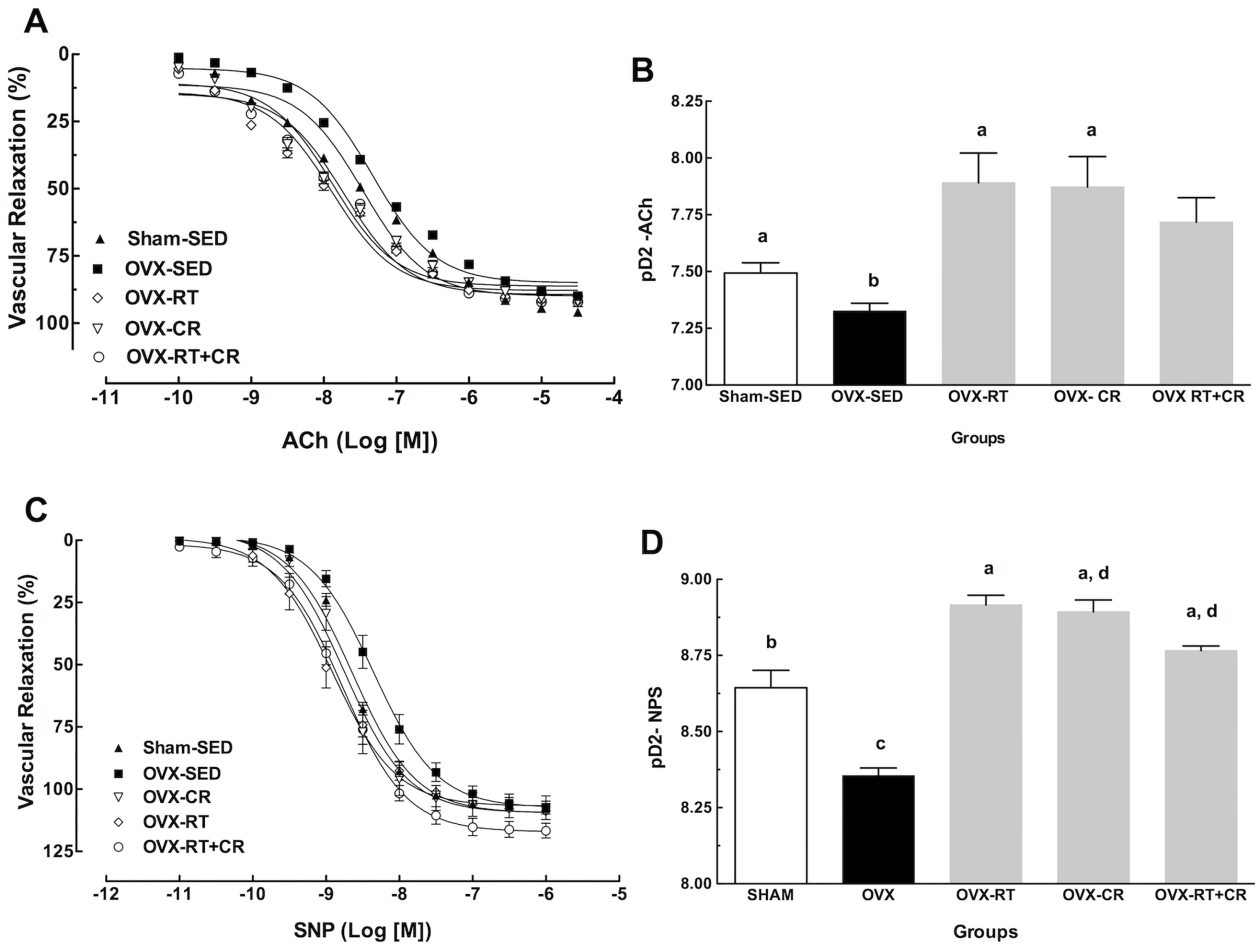
Effects of ovariectomy (OVX), resistance training (RT), caloric restriction (CR) and their association (RT+CR) in aortic rings on endothelium-dependent relaxation induced by acetylcholine (ACh) and endothelium-independent relaxation induced by sodium nitroprusside (SNP). (A) Concentration response curve to ACh; (B) Concentration response curve to SNP; (C) The potency (pD2) relaxant effect by ACh and D) The potency (pD2) relaxant effect by SNP. Superscript letters represent significant difference between groups by a one-way ANOVA with post hoc Tukey test (p<0.05), a ≠ b, b ≠ c, c ≠ d.

OVX decreased the endothelium-dependent relaxation induced by ACh (Pd2, Sham-SED: 7.49±0.04; OVX-SED: 7.32±0.03, p<0.05). RT protocol (OVX-RT) and CR were able to increase endothelium-dependent relaxation induced by ACh when compared to that of OVX-SED (Pd2, OVX-SED: 7.32±0.07; OVX-RT: 7.89±0.13; OVX-CR: 7.87±0.13; p = 0.01, *n* = 08). The association of the RT and CR intervention (RT+CR) did not promote changes when compared to that of the Sham-SED and the OVX groups. No difference was observed between the OVX groups and Sham-SED (Fig 2A and 2B).

Endothelium-independent relaxation induced by sodium nitroprusside (SNP) was impaired in aortic rings of the OVX group (Pd2, Sham-SED: 8.64±0.05; OVX-SED: 8.35±0.02; p = 0.0001, n = 08). The RT protocol (OVX-RT) was able to prevent the impairment promoted by OVX, resulting in an increased independent endothelium relaxation potency (Pd2, Sham-SED: 8.64±0.05; OVX-SED: 8.35±0.02; OVX-RT: 8.91±0.03, p = 0.0001). The same result was observed for the OVX-CR group. CR increased the independent-endothelium relaxation by SNP relative to that of OVX-SED, even improving the relaxation potency when compared to that of Sham-SED (Pd2, Sham-SED: 8.64±0.05; OVX-SED: 8.35±0.02; OVX-CR: 8.89±0.04, p = 0.0001, *n* = 08) (Fig 2C and 2D). Regarding the maximum relaxant effect (Emax), in both dependent (ACh) and independent (SNP) endothelium relaxation, no difference was observed in any of the groups.

### Activity of MMP-2 by gelatin zymography

**Fig 3:**
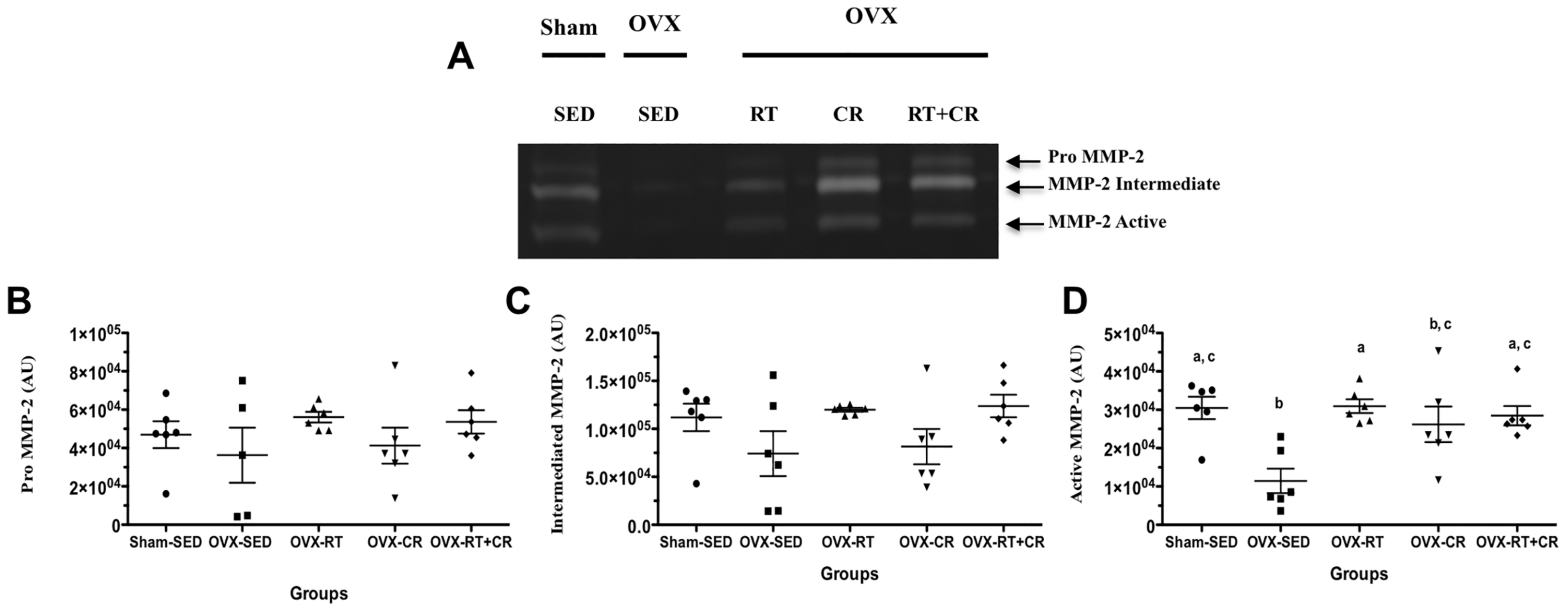
Effects of ovariectomy (OVX), resistance training (RT), caloric restriction (CR) and their association (RT+CR) on pro, intermediate and active matrix metalloproteinase 2 (MMP-2). (A) Representative image of MMP-2 activity by zymography; (B) Pro MMP-2; (C) Values of Intermediate MMP-2; (D) Active MMP-2. Letters superscript represent significant difference between groups by a one-way ANOVA with post hoc Tukey test (p<0.05), a ≠ b, b ≠ c, c ≠ d.

Fig 3A shows a representative activity of MMP-2 by a gelatin zymography assay of the thoracic aorta rings of all groups. Vascular relaxation alterations could be associated with vascular remodeling. RT and CR could attenuate this alteration. To test this hypothesis, aortic samples were submitted to a zymography assay to identify the activity of MMP-2. OVX produced a decreased activity of active matrix metalloproteinase-2 (Active MMP-2) (p<0.05) compared to that of Sham-SED (Fig 3D). However, the interventions of RT and CR and their association (RT+CR) were able to increase the Active MMP-2 activity compared to that of OVX-SED (p = 0.005; p = 0.02; p = 0.03, respectively) and normalize the Active MMP-2 activity compared to that of Sham-SED. No difference was observed in the activity of the pro or intermediate isoforms of MMP-2 in any of the groups (Fig 3B and 3C).

### Morphometric analysis of the aorta wall

**Fig 4:**
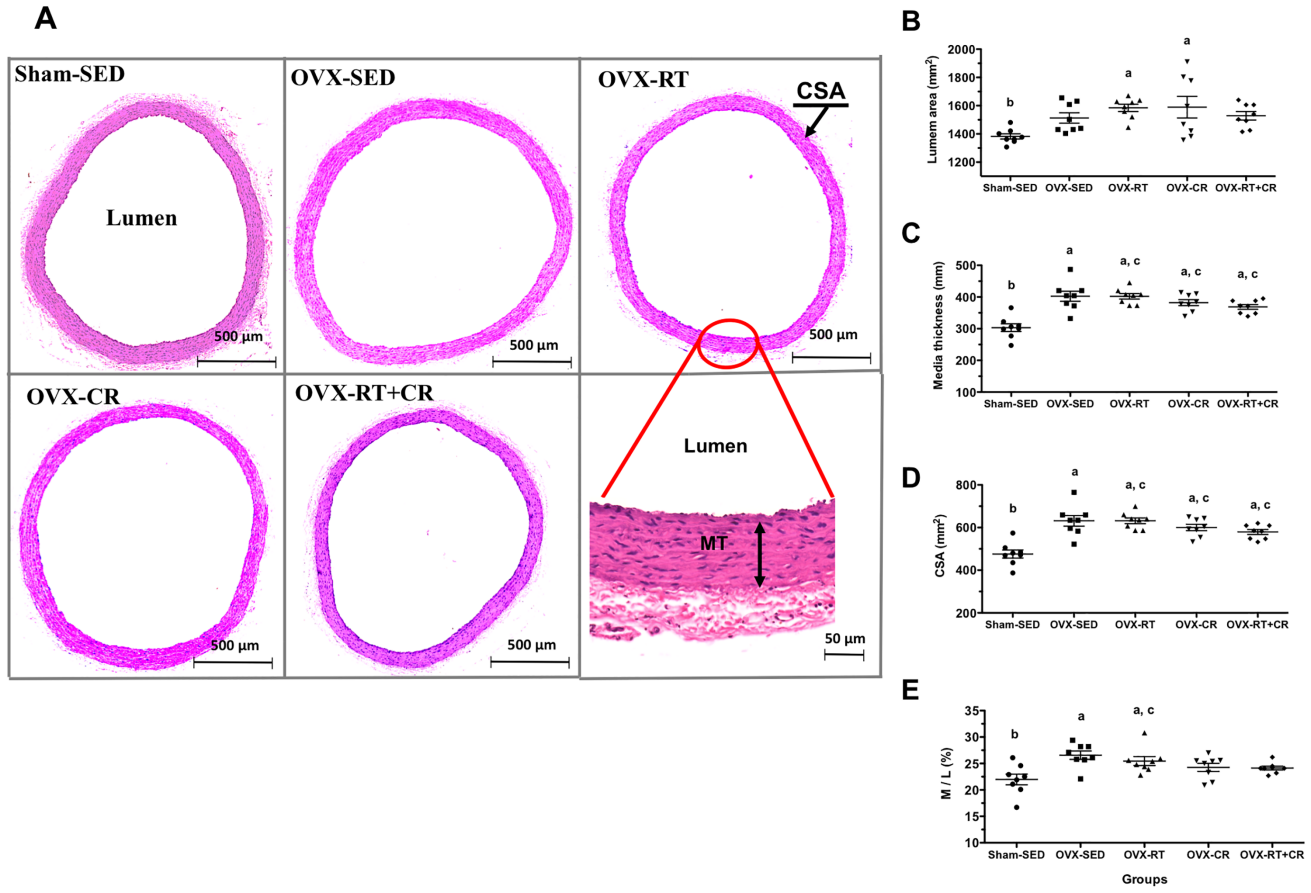
Ovariectomy (OVX), resistance training (RT), caloric restriction (CR) and their association (RT+CR) on structural modifications induced in the thoracic aorta. (A) Representative photomicrography of thoracic aorta of Holtzman rats stained by hematoxylin and eosin (H&E); (B) Values of lumen area (mm^2^); (C) MT, Media thickness (mm); (D) CSA (mm^2^) (Cross-sectional area); and (E) Values of media-to-lumen ratio (M/L), in percentage (%). Superscript letters represent significant difference (p<0.05) between groups by a one-way ANOVA with post hoc Tukey test (all groups), a ≠ b, b ≠ c, c ≠ d.

Fig 4A shows representative figures of all thoracic aorta rings of all groups. OXV increased the lumen diameter (p<0.05) relative to that of Sham-SED. The separate RT and CR protocols showed no alteration of the lumen diameter when compared to that of the Sham-SED and OVX-SED groups (p>0.05) (Fig 4B). On the other hand, the association of RT+CR showed a slight decrease of the lumen diameter, with no change compared to that of Sham-SED and OVX-SED. OVX-SED showed increased media thickness (M) compared to Sham-SED (p<0.05) (Fig 4C). The interventions promoted no modifications relative to those of Sham-SED and OVX-SED (p>0.05). The same results were observed in the CSA, as OVX increased the CSA relative to that of Sham-SED (p<0.05); no difference was observed among the RT, CR, and RT+CR groups (p>0.05) (Fig 4D). The M/L ratio, in % (Fig 4E), was increased by OVX in the SED group when compared to that of Sham-SED (p<0.05). The RT training showed no effects in this variable, maintaining the elevated M/L ratio in OVX animals (OVX-RT) compared to that of Sham-SED (p<0.05). The association of RT and CR protocols was able to mitigate the effect of OVX in the M/L ratio when compared to that of Sham-SED, showing no difference between Sham-SED and OVX-SED (p<0.05).

### Collagen content (%)

**Fig 5:**
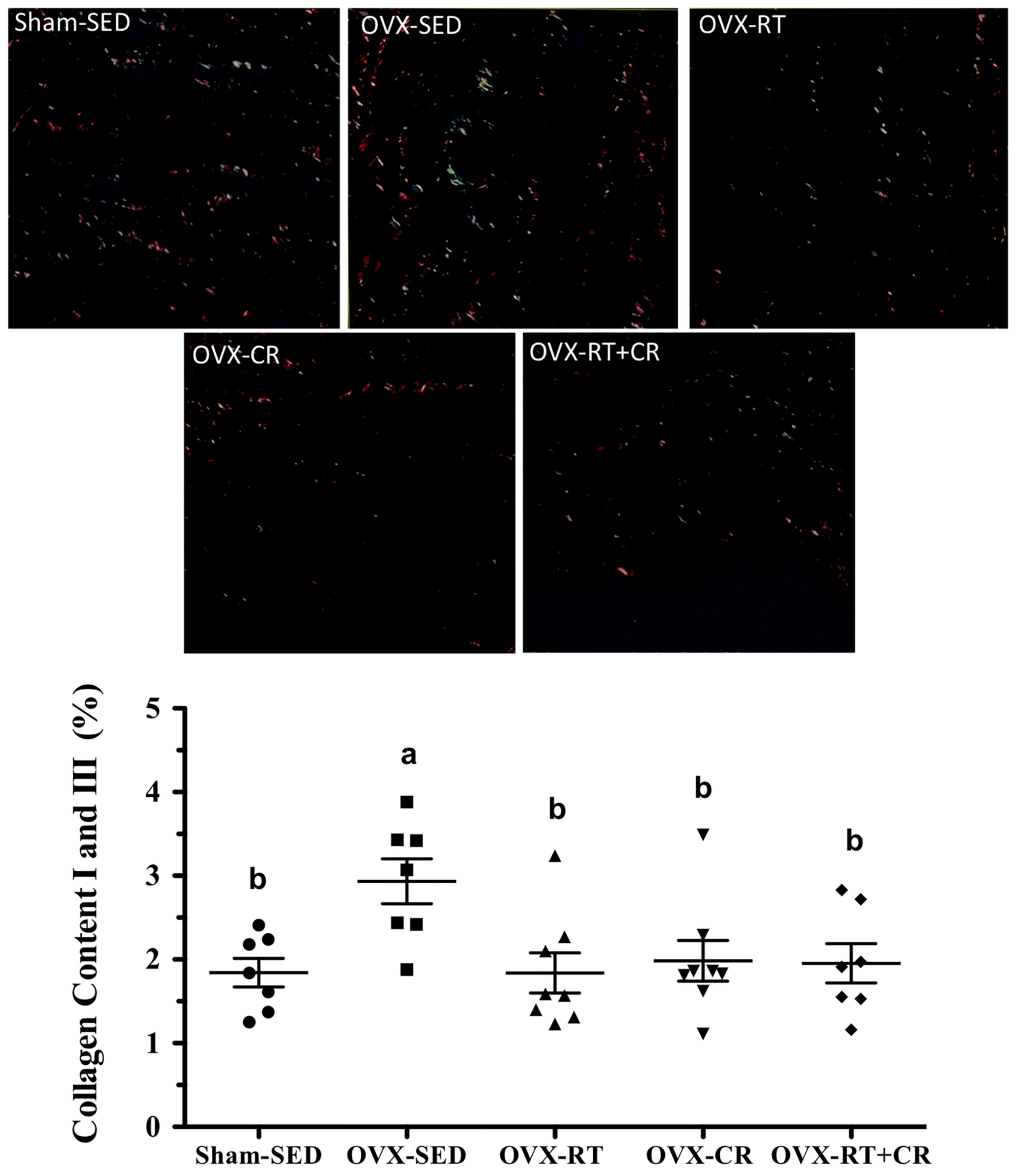
Effects of ovariectomy (OVX), resistance training (RT), caloric restriction (CR) and their association (RT+CR) on percentage of collagen content I and III in the aortic wall, stained with Picrosirius Red. Superscript letters represent significant difference between groups by a one-way ANOVA with post hoc Tukey test (p<0.05), a ≠ b, b ≠ c, c ≠ d.

In the aorta wall of the OVX-SED group, the % of collagen I and III was higher than in Sham-SED (p = 0.02). However, we observed lower % values of collagen content for RT (p = 0.02), CR and their association (p = 0.05, for both) compared to those of OVX-SED, with similar % compared to that of Sham-SED (Fig 5).

### Structure and elastic fibers of aorta walls

**Fig 6:**
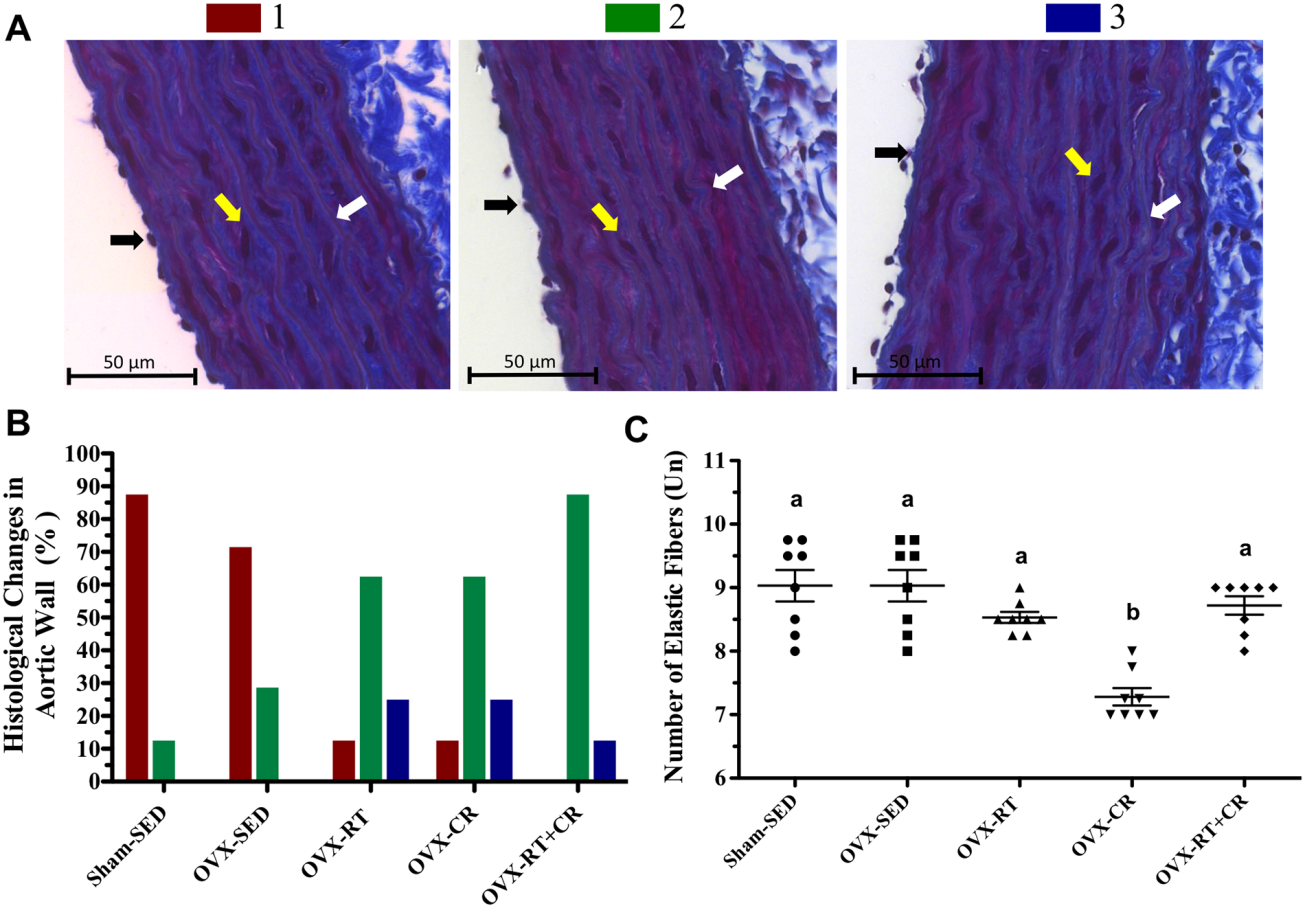
**Effects of ovariectomy (OVX), resistance training (RT), caloric restriction (CR) and their association (RT+CR) on** (A) Histological change in aortic wall structure in categories: Category 1 (Red)- Well-defined endothelial cells, smooth muscle cell without visible changes, linear and parallel elastic fibers, interspersed with smooth muscle cells; Category 2 (Green)- Less endothelial cells, fewer smooth muscle cells, elastic fibers slightly undulated with minimal space and fewer smooth muscle cells between them; Category 3 (Blue)- Few endothelial cells, misshapen structures, altered, wrinkled, thick elastic fibers with minimal space and less smooth muscle between them. The values are presented as percentages (%) with no statistical tests performed. Colors arrows indicate: black- endothelial cells; yellow- smooth muscle cells; and white- elastic fibers; (B) Histological Changes in Aortic Wall (%), and (C) Number of elastic fibers, shown as units, stained with Trichrome-Masson. Letters superscript represent significant difference between groups by a one-way ANOVA with post hoc Tukey test (p<0.05), a ≠ b, b ≠ c, c ≠ d.

OVX produced structural changes in the aorta (Fig 6A and 6B). It was observed that in the OVX-SED group, 71.4% fit into category 1, while the other 28.6% fit into category 2. The Sham-SED showed 87.5% in category 1 and 12.5% in category 2. In the OVX-RT and OVX-CR groups, there was a change in the % within the 3 categories. Both groups presented 12.5% in category 1, 62.5% in 2 and 25% in 3. In the OVX-RT+CR group, it was observed that there was no histological slice that fit into category 1. On the other hand, we observed that 75% were in category 2 and 25% were in category 3.

There was a significant change in the number of elastic fibers (Fig 6C) in the OVX-CR group, with a lower number of this structure than that in the other groups (p<0.05). No other change in the number of elastic fibers was observed among the Sham-SED, OVX-SED, OVX-TR and OVX-TR+CR experimental groups.

### Systolic blood pressure (SBP) and heart rate (HR)

**Fig 7:**
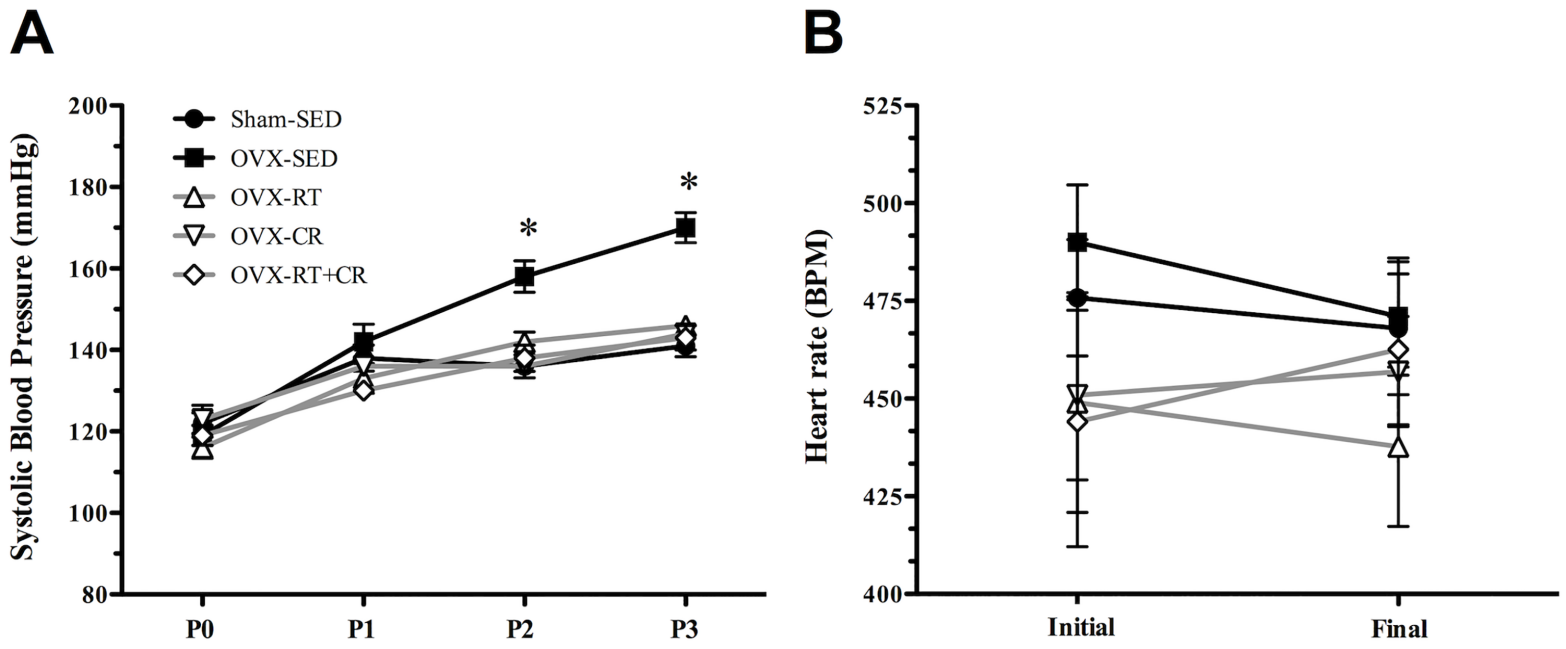
**Effect of ovariectomy (OVX), resistance training (RT), caloric restriction (CR) and their association (RT+CR) in** (A) Evolution of systolic blood pressure (SBP) (mmHg). P0- 24 h before OVX; P1- ten days after OVX and twenty-four hours before starting RT and CR protocols; P2- seven weeks after starting RT and CR protocol; P3- 24 h before euthanasia; and (B) Heart rate in beats per minute (BPM) in initial and final experimental period. Superscript letters represent significant differences between groups by a one-way ANOVA with post hoc Tukey test (p<0.05), a ≠ b, b ≠ c, c ≠ d.

The SBP measurement (mmHg) performed 24 h before the OVX and Sham procedures (P0) and after ten days of OVX (P1) showed no difference among the groups. However, from the seventh week of the experimental period (P2) to the final experimental period (P3), there was a constant increase in SBP in the OVX-SED group compared to that of Sham-SED (p = 0.001). The RT protocol was able to prevent SBP elevation in the OVX-RT group (p = 0.005) compared to that of OVX-SED, keeping the values at a normal level relative to that of the Sham-SED group, as observed in the OVX-CR and the OVX-RT+CR (p = 0.001, for both) groups (Fig 7A). No changes were detected in resting HR at the initial period and after OVX among all the groups. After 13 weeks of RT and CR intervention, alone or both associated, there were also no changes observed in the resting HR among all the groups (Fig 7B).

## Discussion

Both RT and CR have been studied and prescribed to improve vascular function in the absence of estrogen [28, 29], which could prevent some diseases related to menopause, such as hypertension [30–32].

The main finding of our study was that the RT, CR and their combination (RT+CR) are capable of increasing endothelial function in the aortic artery, elevating the activity of MMP-2 with decreased collagen I and III deposition and altering aortic wall remodeling. Taken together, all of these alterations may be responsible for preventing endothelial dysfunction, impairment of MMP-2 activity, increasing collagen I and III deposition and morphological changes in the aortic artery that prevent SBP elevation. Thus, the results of the present study confirm the hypothesis that the interventions, separately or associated, prevent aortic vessel dysfunction, modulating the vasodilator response, stimulating MMP-2 activity, promoting beneficial remodeling and modifying the wall constituents of the aortic vessel, which prevent the SBP increase in an experimental condition of menopause.

Our results showed that OVX impaired the relaxant capacity of the aorta. The RT and CR interventions, in addition to preventing endothelial dysfunction, improve the relaxant capacity of this artery. Endothelial dysfunction is characterized by a reduction of the bioavailability of vasodilators; the dysfunction increases the liberation of vasoconstrictors and has been considered capable of predicting cardiovascular diseases [33]. In endothelial cells, ACh activates the muscarinic receptor inducing NO production by increasing the cytosolic Ca^2+^ concentration, in addition to consecutively activating the endothelial nitric oxide synthase (eNOS) enzyme. The NO produced then migrates to smooth muscle cells, which leads to activation of the soluble guanylyl cyclase (sGC) enzyme, generating cyclic guanosine monophosphate (cGMP), which activates the cGMP-dependent protein kinase (PKG). Such an effect induces a decrease in the cytosolic Ca^2+^ concentration and Ca^2+^ desensitization of actin-myosin contractile system, in vascular smooth muscle cells, promoting vascular relaxation [34]. Various cGMP-independent mechanisms of NO have been reported, such as the direct activation of K^+^ channels [35, 36], as well the activation of Na^+^/K^+^-ATPase pump [36]. Estrogen activates eNOS through the nongenomic pathway PI3K and Akt, promoting NO production [37]. In the absence of estrogen hormone, as in menopause [14] and OVX [15], NO production is impaired, which leads to the onset of endothelial dysfunction.

In the present study, RT and CR increased the endothelial sensitivity to ACh as well as the vasodilation response to SNP. On the other hand, the association of RT and CR in OVX animals (OVX-RT+CR) showed no statistical alteration for the endothelial sensitivity to ACh. However, changes in aortic structures were observed, with 87.5% of the animals in this group were classified in the category 2, and 12.5% in the category 3. For the others OVX groups submitted to both interventions, there was a minimum of animals that presented a percentage, even if small, in category 1 (~ 12%). Category 2 is described in our study as "Less endothelial cells, fewer smooth muscle cells, elastic fibers slightly undulated with minimal space and fewer smooth muscle cells between them". This large structural modification, in the percentage of category 1 to 2, could be responsible for altering the response to ACh, explaining the result observed in the OVX-RT+CR group.

In addition, RT produced an improvement in the vasodilator response to NO, either from a NO donor or from the endothelial production (endogenous to the endothelial cells). Physical exercise, such as RT, increases blood flow during exercise. Increased blood flow mediates the elevation of shear stress in the aorta that could lead to improved endothelium-dependent vasorelaxation [28]. This shear stress potently stimulates the release of NO, PGI2 and endothelium-derived hyperpolarizing factor (EDHF) [38, 39]. The mediated-shear-stress-force in the endothelium activates cGMP formation. NO synthase type III (NOS3) is responsible for the catalysis of the production of NO from the cationic amino acid, L-arginine. The change in the shear force activates NOS via changes in intracellular Ca^2+^ or via a receptor-mediated process. Released NO activates sGC in smooth muscle cells, converting GTP to cGMP. This process is able to activate protein kinase, leading to the inhibition of Ca^2+^ influx into the smooth muscle cell and a decreased calcium-calmodulin stimulation of myosin light chain kinase. The result is a decrease in the phosphorylation of myosin light chains, decreasing smooth muscle tension development and causing vasodilation [40]. Another explanation is that RT enhances vasodilation, probably by improving endothelial function as previously demonstrated [41]. These effects can prevent SBP elevation, as will be further discussed.

Additionally, have been proposed in the literature that reactive oxygen species (ROS) decreases NO and produces peroxynitrite (ONOO^-^) [42]. There are a variety of important ROS producing systems in the vascular wall, among others, the uncoupled eNOS. Uncoupled eNOS produces O_2_^-^ and it reacts with NO resulting in ONOO^-^ generation leading to reduction of endothelial NO production, promoting endothelial dysfunction. The decreased NO e increased ONOO^-^ production promoting endothelial dysfunction, by inactivation of NO by excess O_2_^-^, observed in pathological condition, as hypertension, associated with oxidative stress [42]. On the other hand, CR is able to reduce oxidative stress in the aortic vessel, which is produced by OVX, with limited NO bioavailability leading to endothelial dysfunction. Reduced oxidative stress attenuates the degree to which NO is inactivated by ROS, improving NO bioavailability [13] and endothelium and vascular smooth muscle function, thereby normalizing NO production [29]. CR can modulate endothelial NO activity and expression via the activation of NAD-dependent deacetylase sirtuin-1 (SIRT1), both localized in endothelial cells. SIRT1 deacetylates endothelial nitric oxide synthase (eNOS), stimulating its activity and increasing endothelial NO, leading to normalization of the endothelium function [43]. Thus, RT associated with CR could incorporate the beneficial effects of both interventions, discussed above, contributing to improving the aorta vasorelaxation in OVX animals. These factors could explain the improvement in endothelium and vascular smooth muscle function, which may be related to the maintenance of the SBP values in rats submitted to OVX. Anyway this may have happened in our study.

Insulin resistance, observed in OVX animals and humans, can promote endothelial dysfunction. The role of insulin resistance in endothelial dysfunction has been previously elegantly revised [3, 44]. Therefore, 12 weeks of aerobic training by treadmill running exercise with 50% CR promoted improvements in insulin sensitivity in OVX Wistar rats [45]. To date, we did not find studies in the literature that reported researchers’ use of combined RT and CR in OVX rats. On the other hand, we found an elegant study showing that endurance training is able to ameliorate insulin sensitivity in OVX rodents after eight weeks of training [46]. Isolated CR (35%) ameliorated insulin resistance in OVX Sprague-Dawley rats after a 12-week experimental period [47], similar to our results. Thus, OVX can promote endothelial dysfunction by altering the NO production and by generating insulin resistance that also contributed to the impairment of the relaxant capacity of aorta. These factors together can elevate SBP leading to hypertension, a condition that was prevented by RT and CR, according to our results.

Endothelial dysfunction and insulin resistance can modify not only the relaxant capacity of the aorta but also the amount of elastic and collagen fibers that are important to provide adequate mechanical properties to the wall of this artery. Alterations of these constituents, such as increased collagen and decreased elastic fibers, compromise the capacity of vessel distension. The elastic fiber can be degraded by MMP-2, resulting in a change in ECM homeostasis modification [48], collagen deposition and arterial stiffness [2], as observed in the present study. On the other hand, the studied interventions promoted increased MMP-2 activity. Recently, interesting works revised the MMPs action in aortic tissue [48]. Lam and colleagues [6] observed that in OVX animals, MMP-2 activity had a marked reduction in the thoracic aorta with a significant increase in collagen accumulation. Those effects were reverted by estrogen replacement. Thus, the decrease in MMP-2 activity leads to vascular collagen accumulation. Vascular collagen accumulation is involved in the elevation of blood pressure caused by OVX. Similar results were found in our study. The positive effects of MMP-2 in the aorta, observed in the RT and CR experimental groups, provides a modicum of understanding of SBP control in OVX rats, as shown in the present study. However, controversial results were found in a study with OVX rodents that received estrogen replacement, in which MMP-2 activity was elevated, showing an interaction of estrogen hormone with MMP-2 activity in the aorta [49]. Interestingly, in present the work, the same results were observed when OVX animals were submitted to RT and CR protocols.

These controversial results can be found even in clinical hypertension trials, indicating a decreased MMP-2 protein expression in plasma from patients with hypertension [8]. Another study showed similar results, demonstrating the depression of MMP-2 activity in plasma concentrations of patients with essential hypertension [50]. A clinical study in 44 hypertensive patients and 44 controls found that the plasma concentrations and MMP-2 activity are increased in hypertensive patients, which may reflect abnormal ECM metabolism [33]. The high SBP, endothelial dysfunction, and altered MMP-2 activity in collagen content can promote artery wall remodeling.

OVX can cause vessel remodeling characterized as outward hypertrophic remodeling [26], as observed in the increase in the constituents of tunica media (M), analyzed by CSA and lumen area (L). In our study, only the RT protocol influenced the M/L ratio. RT+CR also produced the same hypertrophic outward remodeling, as well as the separate RT and CR. This fact is likely a result of the effect of RT in increased aorta blood flow, which promotes the signal for an increase in the constituents of tunica media and the consequential increase in CSA [12], which could generate a vessel hypertrophic outward remodeling [26].

In the literature, we found that rats submitted to aerobic exercise presented with reduced wall thickness and with increased density of smooth muscle cell nuclei per tunica media unit area [12]. CR is able to attenuate oxidative stress, elevate M constituents and enlarge the aorta wall [13, 51]. However, exercise and diet separately or combined (RT+CR) can statistically equilibrate the M/L ratio in OVX animals compared to the ratio in the Sham-SED animals. Changes resulting from OVX can lead to endothelial dysfunction and a decrease in the response capacity and relaxation of the aortic vessel, which may lead to increased CSA, as discussed above. A study conducted with OVX spontaneously hypertensive rats (SHR) subjected to aerobic exercise [52] found similar results in the aorta thickness of sedentary and aerobically exercised OVX animals.

However, rodent type, exercise protocols, and morphological assay differ from the present study. In the literature, a work conducted to investigate the effect of estrogen replacement on arterial distensibility and endothelial function in Sprague-Dawley rats observed no changes in aorta histomorphometry after 20 days of OVX [15]. Compared to our results, it is clear that aorta histomorphometry is time dependent on ovarian hormone depletion. Notwithstanding, morphological changes can be related to functional changes and endothelial dysfunction [26].

The emergence of insulin resistance, associated with decreased vascular reactivity and lowered activity of MMP-2, which leads to vascular collagen accumulation and changes in elastic fibers in the aorta, may be involved in the elevation of SBP caused by OVX. Both RT and CR influenced the increase in MMP-2 activity, which occurred concomitantly with a decrease in collagen deposition. However, we found interesting changes in the structures of the aortic vessel in our study, in morphologic features, collagen content and elastic fiber properties of the aortic wall.

In relation to SBP, it can be altered by the autonomous nervous system, which is observed in OVX animals. Possible changes in this system can be observed by heart rate (HR) and SBP values. In OVX rats, the sympathetic tone rises, with a parasympathetic decrease. This change determines higher HR and consequent elevation of SBP [11]. Exercise and CR are ways of modulating autonomic tonus, decreasing the sympathetic action and elevating a parasympathetic action in rest conditions, promoting a decrease in SBP [11, 53]. However, in our study, OVX did not alter HR values, as was observed in all interventions. Thus, we strongly believe that elevated SBP and the preventive effects of exercise and diet may have been caused by other mechanisms, such as modulating vascular response (vascular reactivity), as well as remodeling of the aorta. To test this hypothesis, the vascular reactivity was performed on aortic rings in order to identify a possible endothelial dysfunction as a result of OVX and possible preventive effects of exercise and diet in this condition, as discussed.

In summary, OVX (estrogen deprivation) changes the responsiveness of the aortic artery, which may be indicative of endothelial dysfunction, and changes the remodeling, morphology and content of collagen and elastic fibers of the aorta wall. These changes can contribute to elevated SBP, leading the organic system to hypertension. This evolution of SBP may not be due to the alteration of autonomic tonus, observed by the maintenance of the HR values. The second interesting result was that the exercise and diet interventions, even separately, promoted a better responsiveness of the aortic artery due to the prevention of the onset of endothelial dysfunction. In addition, these interventions promoted a beneficial remodeling of the aortic wall, modulating both collagen deposition and elastic fibers, and prevented the onset of hypertension, exerting effects that maintained SBP at normal levels. The limitation of our study is not to have evaluated the estrous cycle of the animals studied for the hemodynamic evaluation.

We can also note that both interventions helped in the prevention of endothelial dysfunction in the aortic artery and SBP increases in the proposed model. Furthermore, our study found an inductive model of SBP elevation dependent on ovarian function without genetic manipulation in rodents. Thus, we believe that the OVX is a useful animal model for studying vascular remodeling and endothelial dysfunction mediated by the absence of ovarian hormones in rodents.

## Conclusion

The present study showed that the effects of resistance training and caloric restriction, performed separately or combined, were efficient interventions to prevent OVX-induced endothelial dysfunction by altering and ameliorating endothelial function, elevating MMP-2 activity, preventing insulin resistance, decreasing collagen deposition and promoting beneficial aortic wall remodeling, which together maintain the normal values of SBP in OVX rats.

## Supporting information

S1 Dataset Data for Table 1. Values of body mass, U/TL ratio, kITT test and maximal workload.
(DOCX)

S2 Dataset Values of endothelium-dependent relaxation induced by acetylcholine (ACh) and endothelium-independent relaxation induced by sodium nitroprusside (SNP) in aortic rings. (DOCX)

S3 Dataset Values of pro, intermediate and active matrix metalloproteinase 2 (MMP-2) activity. (DOCX)

S4 Dataset Structural modifications induced in the thoracic aorta. Stained by hematoxylin and eosin (H&E); Values of lumen area (mm^2^); MT, Media thickness (mm); CSA (mm^2^) (Cross-sectional area); and Values of media-to-lumen ratio (M/L), in percentage (%).
(DOCX)

S5 Dataset Percentage of collagen content I and III in the aortic wall, stained with Picrosirius Red. (DOCX)

S6 Dataset Histological change in aortic wall structure in categories 1, 2 and 3. Number of elastic fibers, shown as units, stained with Trichrome-Masson.
(DOCX)

S7 Dataset Evolution of systolic blood pressure (SBP) (mmHg). P0- 24 h before OVX; P1- ten days after OVX and twenty-four hours before starting RT and CR protocols; P2- seven weeks after starting RT and CR protocol; P3- 24 h before euthanasia; and Heart rate in beats per minute (BPM) in initial and final experimental period.
(DOCX)

S8 Dataset Raw data. (XLSX)
