# Supplementary material for: Resistance training and caloric restriction prevent systolic blood pressure rise by improving the nitric oxide effect on smooth muscle and morphological changes in the aorta of ovariectomized rats
Source: PLoS One. 2018 Aug 22;13(8):e0201843. doi: 10.1371/journal.pone.0201843 (PMC6104970; doi:10.1371/journal.pone.0201843)
Supplement: S1 Dataset — Values of body mass, U/TL ratio, kITT test and maximal workload. (DOCX) [file pone.0201843.s001.docx]

# Values of body mass, U/TL ratio, kITT test and maximal workload.
