# Supplementary material for: Resistance training and caloric restriction prevent systolic blood pressure rise by improving the nitric oxide effect on smooth muscle and morphological changes in the aorta of ovariectomized rats
Source: PLoS One. 2018 Aug 22;13(8):e0201843. doi: 10.1371/journal.pone.0201843 (PMC6104970; doi:10.1371/journal.pone.0201843)
Supplement: S4 Dataset — Stained by hematoxylin and eosin (H&E); Values of lumen area (mm2); MT, Media thickness (mm); CSA (mm2) (Cross-sectional area); and Values of media-to-lumen ratio (M/L), in percentage (%). (DOCX) [file pone.0201843.s004.docx]

# Structural modifications induced in the thoracic aorta. Stained by hematoxylin and eosin (H&E); Values of lumen area (mm^2^); MT, Media thickness (mm); CSA (mm^2^) (Cross-sectional area); and Values of media-to-lumen ratio (M/L), in percentage (%).

#

# 
