# Supplementary material for: Resistance training and caloric restriction prevent systolic blood pressure rise by improving the nitric oxide effect on smooth muscle and morphological changes in the aorta of ovariectomized rats
Source: PLoS One. 2018 Aug 22;13(8):e0201843. doi: 10.1371/journal.pone.0201843 (PMC6104970; doi:10.1371/journal.pone.0201843)
Supplement: S6 Dataset — Number of elastic fibers, shown as units, stained with Trichrome-Masson. (DOCX) [file pone.0201843.s006.docx]

# Histological change in aortic wall structure in categories 1, 2 and 3. Number of elastic fibers, shown as units, stained with Trichrome-Masson.

# 
