# Supplementary material for: Resistance training and caloric restriction prevent systolic blood pressure rise by improving the nitric oxide effect on smooth muscle and morphological changes in the aorta of ovariectomized rats
Source: PLoS One. 2018 Aug 22;13(8):e0201843. doi: 10.1371/journal.pone.0201843 (PMC6104970; doi:10.1371/journal.pone.0201843)
Supplement: S7 Dataset — P0- 24 h before OVX; P1- ten days after OVX and twenty-four hours before starting RT and CR protocols; P2- seven weeks after starting RT and CR protocol; P3- 24 h before euthanasia; and Heart rate in beats per minute (BPM) in initial and final experimental period. (DOCX) [file pone.0201843.s007.docx]

# Evolution of systolic blood pressure (SBP) (mmHg). P0- 24 h before OVX; P1- ten days after OVX and twenty-four hours before starting RT and CR protocols; P2- seven weeks after starting RT and CR protocol; P3- 24 h before euthanasia; and Heart rate in beats per minute (BPM) in initial and final experimental period.

# 
